# Regulation of fungal decomposition at single-cell level

**DOI:** 10.1038/s41396-019-0583-9

**Published:** 2020-01-02

**Authors:** Michiel Op De Beeck, Carl Troein, Syahril Siregar, Luigi Gentile, Giuseppe Abbondanza, Carsten Peterson, Per Persson, Anders Tunlid

**Affiliations:** 1 Department of Biology, Microbial Ecology Group, Lund University, Ecology Building, SE-223 62 Lund, Sweden; 2 Department of Astronomy and Theoretical Physics, Computational Biology and Biological Physics, Lund University, Sölvegatan 14A, SE-223 62 Lund, Sweden; 3 Department of Chemistry and CSGI, University of Bari Aldo Moro, IT- 701 21 Bari, Italy; 4 Department of Physics, Synchrotron Radiation Research, Lund University, SE- 223 62 Lund, Sweden; 5 Centre for Environmental and Climate Research (CEC), Lund University, Ecology Building, SE-223 62 Lund, Sweden

**Keywords:** Biogeochemistry, Biogeochemistry, Biogeochemistry, Microbial ecology, Fungal ecology

## Abstract

Filamentous fungi play a key role as decomposers in Earth’s nutrient cycles. In soils, substrates are heterogeneously distributed in microenvironments. Hence, individual hyphae of a mycelium may experience very different environmental conditions simultaneously. In the current work, we investigated how fungi cope with local environmental variations at single-cell level. We developed a method based on infrared spectroscopy that allows the direct, in-situ chemical imaging of the decomposition activity of individual hyphal tips. Colonies of the ectomycorrhizal Basidiomycete *Paxillus involutus* were grown on liquid media, while parts of colonies were allowed to colonize lignin patches. Oxidative decomposition of lignin by individual hyphae growing under different conditions was followed for a period of seven days. We identified two sub-populations of hyphal tips: one with low decomposition activity and one with much higher activity. Active cells secreted more extracellular polymeric substances and oxidized lignin more strongly. The ratio of active to inactive hyphae strongly depended on the environmental conditions in lignin patches, but was further mediated by the decomposition activity of entire mycelia. Phenotypic heterogeneity occurring between genetically identical hyphal tips may be an important strategy for filamentous fungi to cope with heterogeneous and constantly changing soil environments.

## Introduction

Filamentous fungi play a key role in both the organic and inorganic nutrient cycles of terrestrial ecosystems through decomposition, respiration and the de novo production of biomass and microbially derived compounds such as secondary metabolites [1]. Basidiomycetes in particular, play an important role in these nutrient cycles as they possess the unique ability to decompose lignocellulose from woody plants, recycling much of the biomass synthesized by these plants [2]. Hydrolytic and/or oxidative decomposition mechanisms responsible for decomposition of lignocellulose can be found in both saprotrophic and symbiotic (i.e., ectomycorrhizal, ECM) Basidiomycetes [3, 4]. Unlike saprotrophic fungi, ECM fungi do not decompose lignocellulose to acquire metabolic carbon, but instead forage for other nutrients, such as nitrogen and phosphorus, which are often complexed with or embedded in lignocellulosic material in soils [3, 4].

Most soil-borne Basidiomycetes are multicellular filamentous fungi that grow through apical growth, forming elongated chains of isogenic cells that may be separated from each other through internal cross walls, called septa. Septa may be left open in some cells, whereas in others, septa can be closed by specialized organelles such as parenthesomes [5]. At the apical end of hyphae (hyphal tips), oxidative and hydrolytic enzymes and secondary metabolites are secreted, causing decomposition of substrates and the release of nutrients [6, 7]. Through their almost indeterminate growth, Basidiomycetes can occupy anywhere from a few cubic millimeters to several cubic meters of soil, sometimes forming large fungal webs [8]. Hence, different parts of a mycelium may be exposed to very different environments simultaneously. Fungi have developed several mechanisms to overcome the challenges of living in heterogeneous soil environments. One well-studied mechanism in this respect is the translocation of organic and mineral nutrients throughout mycelia. This way, nutrients can be supplied to support growth of the entire mycelium, even if certain parts of the mycelium are growing in nutrient-poor soil patches. Nutrient translocation throughout mycelia has been studied in detail for both ECM fungi [9, 10] and saprotrophic fungi [11, 12], which can transport nutrients over large distances (decimeters to meters) through specialized hyphal structures such as cords or rhizomorphs. Fungi can also efficiently re-structure their mycelia, recycling nutrients contained in hyphae that are no longer needed (e.g., hyphae present in nutrient-depleted parts of soils) through autophagy, and they can produce new hyphae in nutrient-rich parts of soils to optimize nutrient uptake [13]. Despite the existence of mechanisms that even out variations in environmental conditions experienced by mycelia, genetically identical cells in mycelia may still display large variations in decomposition activity [14]. It is unclear why this metabolic heterogeneity in mycelia exists. It is also unclear how environmental factors affect this metabolic heterogeneity and how the decomposition activity of individual fungal cells relate to the overall decomposition activity of entire mycelia.

Furthermore, insight in the interactions between fungi, organic matter, and the physical and chemical conditions that occur at the very interface between hyphal cells and their immediate microenvironments is crucial for understanding the processes that control the decomposition of soil organic matter. Indeed, over the past few decades, it has become clear that soil physical and chemical parameters, traditionally measured in bulk samples, do not correlate well with microbial activity, and the need for soil microbial processes to be studied at scales that are more relevant to the microorganisms involved has been put forward [15]. An explanation for the slow progress in characterizing biogeochemical processes at the scale of microorganisms is the lack of appropriate methods. Current analytical techniques that allow microscopic imaging of chemical interactions between microbes and their environment require the sample compartment to be under vacuum and/or provide limited chemical information [16–22].

The main aims of the current study were therefore (i) to develop a nondestructive method that enables the in-situ spatial characterization of chemical interactions between individual fungal hyphal tips and their environments and (ii) to examine how environmental conditions in local organic patches colonized by parts of mycelia affect the decomposition activity of individual hyphae within the patches and how this activity is influenced by the decomposition activity of entire fungal mycelia.

**Fig. 1:**
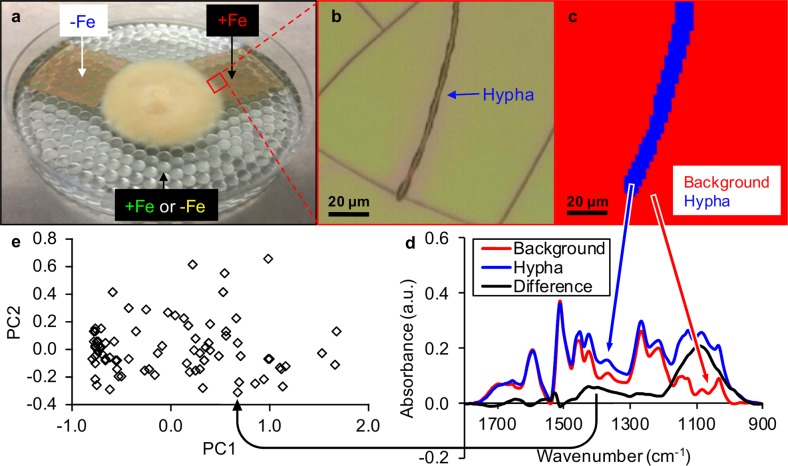
Schematic overview of the experimental set-up and data collection routine used in chemical imaging of the oxidative decomposition of lignin by individual hyphal tips of *Paxillus involutus*. Starting from the top left image and following clockwise: **a** Schematic overview of the experimental set-up where *P. involutus* cultures were grown on liquid media and allowed to colonize two lignin patches (one patch containing no iron, “−Fe”, and one patch containing 1 mg Fe g^−1^ lignin, “+Fe”). The liquid medium either contained 74 µM FeCl_3_•6H_2_O (experiment 1, “+Fe”, resulting in mycelia with high oxidative decomposition activity) or no iron (experiment 2, “−Fe”, resulting in mycelia with low oxidative decomposition activity). **b** Six individual hyphal tips were imaged per lignin patch, with the white light image of only one hyphal tip shown in this figure as an example. **c** Pixels in hyperspectral images were clustered according to chemical similarity using in-house built software. **d** Subsequently, the average spectrum of pixels not affected by decomposition (red, background) was subtracted from the average spectrum of pixels affected by oxidative decomposition (blue, hypha), resulting in a single difference spectrum (black) for each image. This difference spectrum represents the average net effect of metabolic activity (including secretion of metabolic products and oxidative decomposition of the lignin substrate) caused by a single hypha. Absorbance values are expressed in arbitrary units (a.u.). **e** Finally, similarities between difference spectra were visualized using principal component analyses and statistically compared across incubation times, lignin patches and experiments using permutational multivariate analysis of variance.

To address these questions, we developed a nondestructive procedure using infrared (IR) spectroscopy that allows the direct, in-situ, chemical imaging of the decomposition activity of a large number of individual hyphal tips at a micrometer scale. This technique was used to investigate the oxidative decomposition of lignin by individual hyphae of the ECM Basidiomycete *Paxillus involutus*. *P. involutus* was grown axenically on liquid media, while parts of the mycelia were allowed to colonize discrete patches of lignin (Fig. 1), one of the main constituents of soil organic matter [23, 24]. The molecular mechanisms by which *P. involutus* decomposes soil organic matter and the environmental cues that control these mechanisms have been studied in detail [25, 26]. Spectroscopic analyses have shown that *P. involutus* is capable of oxidizing lignin using hydroxyl radicals (^•^OH) generated by an extracellular Fenton reaction (Fe^2+^ +H_2_O_2_+H^+^ → Fe^3+^ +^•^OH+H_2_O) in a mechanism similar to that of saprotrophic brown-rot fungi [27]. Oxidation by ^•^OH does not result in an extensive degradation of lignin but rather in specific chemical modifications of side chains in lignin [27]. The Fenton reaction requires the presence of ferrous iron (Fe^2+^) and the production of ^•^OH is strongly induced when the fungus switches from using an inorganic (NH_4_^+^) to an organic nitrogen source (Bovine Serum Albumin, BSA) [25]. Such detailed knowledge of the mechanisms and nutritional factors that control the oxidative decomposition system is a necessary prerequisite for addressing the aims of the current study.

From the presented data, we conclude that the local environmental conditions experienced by parts of mycelia colonizing lignin patches strongly affect the oxidative decomposition activity at single-cell level, but this effect is further mediated by the overall oxidative decomposition activity of mycelia. A subpopulation of more actively decomposing hyphae could be distinguished from a subpopulation of less active hyphae despite a large variation in decomposition activity observed in hyphae from the same mycelium. We also demonstrate that the decomposition activity of a fungal mycelium can result from an increased number of actively decomposing hyphae rather than an overall increase in activity of all the hyphae in a mycelium. These results highlight the importance of taking into consideration the local microscale environment as experienced by parts of mycelia when studying fungal decomposition. The developed method provides a suitable platform to acquire detailed chemical information on large numbers of individual cells in a nondestructive way and with micrometer spatial resolution, allowing the study of chemical interactions at the interface between microbial cells and their immediate environments.

## Materials and methods

### Fungal strain and growth conditions

*P. involutus* (Batsch) Fr. strain ATCC 200175 (Manassas, VA, USA) was maintained on modified Fries medium [28]. The medium contained 3.74 mM NH_4_Cl, 0.41 mM MgSO_4_•7H_2_O, 0.22 mM KH_2_PO_4_, 0.18 mM CaCl_2_•2H_2_O, 0.34 mM NaCl, 1.34 mM KCl, 0.24 mM H_3_BO_3_, 20 μM ZnSO_4_•7H_2_O, 5.01 μM CuSO_4_•5H_2_O, 50.29 μM MnSO_4_•H_2_O, 0.16 μM (NH_4_)_6_Mo_7_O_24_•7H_2_O, 74 μM FeCl_3_•6H_2_O, 33.30 mM d-glucose, 55.51 μM myo-inositol, 0.30 μM thiamine•HCl, 0.10 μM biotin, 0.59 μM pyridoxine, 0.27 µM riboflavin, 0.82 μM nicotinamide, 0.73 μM *p*-aminobenzoic acid, and 0.46 μM Ca-pantothenate. The pH of the medium was adjusted to 4.8 using HCl and KOH. Agar was added to 1% (w/v). Cultures were grown at 21 °C in the dark.

For experiments, fourteen *P. involutus* cultures were grown on a monolayer of glass beads and liquid modified Fries medium, amended with 5 µM of BSA and 100 µM coumarin. BSA was added to the growth medium as it was shown to induce ^•^OH production by *P. involutus* [25]. Coumarin was added to detect ^•^OH [25]. No adverse effects of 100 µM coumarin on the growth of *P. involutus* have been observed in preliminary experiments (data not shown). All fourteen cultures were first pre-grown on modified Fries medium for nine days. After these nine days, the liquid medium was removed and replaced with modified Fries medium without NH_4_Cl and cultures were incubated for another 24 h to induce nitrogen starvation [29]. Finally, this medium was removed and replaced with full liquid Fries medium, including BSA and coumarin. In the first experiment (seven of the fourteen cultures), 74 μM FeCl_3_•6H_2_O was included in the final liquid growth medium to induce the oxidative decomposition activity of mycelia. In the second experiment (the other seven cultures), iron was omitted from the final liquid growth medium to inhibit the oxidative decomposition activity of mycelia. Together with the introduction of the final liquid growth media, two lignin patches were placed in front of each of the fourteen growing colonies (Fig. 1). One patch contained pure lignin, while the other patch contained lignin amended with ferrihydrite. After 24 h of incubation, one colony of each experiment was investigated, while the other twelve colonies were left to grow undisturbed. After 48 h of incubation, another set of colonies was investigated, while the remaining ten colonies were left undisturbed. This sampling scheme was continued for a period of seven days. For each of the investigated colonies, six hyphal tips growing on the patch containing only lignin were imaged and used as biological replicates. The same was done for six hyphal tips growing on ferrihydrite amended lignin patches for each of the colonies. The experimental design and data collection routine was illustrated in Fig. 1a for one of the colonies. In the white light image shown in Fig. 1b, cracks can be observed in the lignin patch. This is typical for the relatively thick (hundreds of nanometers) patches produced in the current study and result from internal stresses during drying of the lignin patches [30]. These cracks introduce physical heterogeneity in the lignin patches, but not chemical heterogeneity, as can be seen from the uniformity in the background of the hyperspectral cluster image in Fig. 1c.

### Production of lignin patches

Kraft lignin (product number 370959, Merck Group, Darmstadt, Germany) was dissolved in 25% NH_4_OH in a ratio of 16% w/w for a minimum of 24 h under constant stirring. The solution was subsequently filter sterilized through 0.45 µm polyether sulfone filters. For patches containing ferrihydrite in addition to lignin, 16% v/v of a ferrihydrite suspension (containing 1 g of Fe per ml suspension) was filter sterilized and added to the dissolved lignin, resulting in a final ratio of 1 mg Fe g^−1^ lignin. This lignin-ferrihydrite mixture was stirred for another 10 min before being filter sterilized and spin-coated. Ferrihydrite was produced as described in [31]. One milliliter aliquots of the sterile lignin solutions were subsequently spin-coated on sterile microscope slides (2.5 cm by 2.5 cm) coated with a 10 nm reflective gold layer (Platypus Technologies, Madison, Wisconsin, USA) at 2000 rpm for 60 s under aseptic conditions. Lignin patches were further air dried in a laminar air flow hood for 10 min before being placed in front of the growing *P. involutus* colonies.

The thickness and surface roughness of lignin patches were analyzed using an atomic force microscope in noncontact mode (AFM-Park XE 100, Suwon, South Korea). The thickness of patches was measured by cutting away a corner of the deposited lignin patches with a scalpel and measuring the difference in height between the exposed gold surface and the remainder of the patch for five patches containing lignin only and five patches containing lignin and ferrihydrite that were not colonized by *P. involutus*. Patch thickness was measured to be 736 ± 65 nm (standard deviation) for lignin patches without ferrihydrite and 753 ± 83 nm for lignin patches with ferrihydrite.

To confirm that no ammonium was left in the lignin patches after drying, five patches containing lignin only and five patches containing lignin and ferrihydrite that were not colonized by *P. involutus* were analyzed using X-ray photoelectron spectroscopy (XPS). XPS was performed on an X-Tool automated XPS microprobe (Physical Electronics, Chanhassen, Minnesota, USA). No detectable amounts of nitrogen were found in any of the lignin patches (data not shown).

### IR spectroscopy, hyperspectral data processing, and statistical analyses

IR spectroscopy images were collected in reflection mode on a Bruker Hyperion 3000 microscope coupled to a Bruker Vertex v70 spectrometer containing a globar mid-infrared source (Bruker Corporation, Billerica, Massachusetts, USA). A liquid nitrogen cooled focal plane-array detector (with 64 by 64 detector elements) and a 15x Cassegrain objective were used. Spectral resolution was 4 cm^−1^. Spectra were collected as 1000 averaged scans. A bare reflective gold surface was used as background.

Hyperspectral images were processed with in-house developed software. Spectral contributions of rotational vibrations of atmospheric water vapor and CO_2_ gas and spectral contributions due to Mie scattering were minimized using this software. Spectra were cut to keep only the fingerprint region between 900 cm^−1^ and 1800 cm^−1^ and rubberband baseline corrected [32]. Purest concentrations and purest spectra were estimated using the simple-to-use interactive self-modeling mixture analysis algorithm [33, 34] and the multivariate-curve resolution alternating least squares algorithm [35]. K-means clustering with eight clusters was finally used to cluster pixels with chemically similar spectra. Clusters corresponding to areas affected by fungal metabolic activity were grouped and averaged. The same was done for clusters corresponding to the background (Fig. 1c). The average background spectrum was subtracted from the average hypha spectrum, resulting in a difference spectrum (Fig. 1d). Such a difference spectrum was calculated for each hyperspectral image and used in further statistical analyses (Fig. 1e).

For both experiments, a total of 84 hyperspectral images were collected (seven days x two patch types x six hyphae per lignin patch), for a total of 168 images. Difference spectra were compared across incubation times, patch types and experiments using permutational multivariate analysis of variance based on a Euclidean distance matrix and 1000 permutations in R 3.6.0 [36]. Principal component (PC) analysis was used to reduce the dimensionality of the difference spectra using the prcomp function in R. Difference spectra were clustered using model-based clustering with the mclust package in R and the first two PCs. Fisher’s exact tests were performed using the fisher.test function in R.

## Results

### Effect of the presence or absence of iron in liquid media on the overall oxidative decomposition activity of *P. involutus* colonies

The effect of the presence or absence of iron in liquid media on the overall oxidative decomposition activity (measured as production of ^•^OH) of *P. involutus* colonies can be found in Supplementary Fig. 1a. From this figure, it is clear that the oxidative decomposition activity of *P. involutus* colonies changed with incubation time and that these changes differed between the two experiments outlined in Fig. 1. The ^•^OH concentrations kept increasing every day under both conditions, but much higher concentrations of ^•^OH were produced in the presence of iron. Hence, the presence (experiment 1) or absence (experiment 2) of iron in liquid media resulted in colonies with high or low overall oxidative decomposition activity, respectively. When iron was present in the liquid media, the reduction of Fe^3+^ to Fe^2+^ increased steadily until reaching a plateau on day five (Supplementary Fig. 1b). No iron reduction was detected when iron was absent from liquid media (Supplementary Fig. 1b).

### Variations in lignin modification between individual hyphae of *P. involutus*

**Fig. 2:**
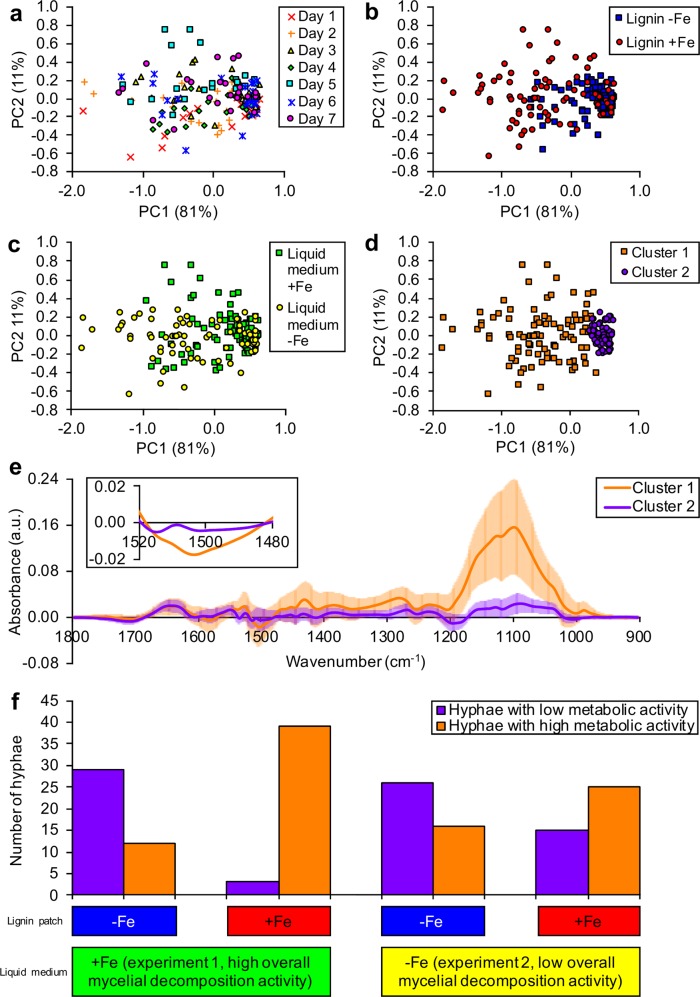
Regulation of the metabolic activity of individual hyphal tips of *P. involutus* by the presence or absence of iron in lignin patches or liquid incubation media. Principal component analysis was used to visually compare difference spectra across incubation times, patch types and experiments. **a** The first two principal components of difference spectra marked according to incubation time (days). **b** The first two principal components of difference spectra marked according to lignin patch type. “Lignin −Fe” denotes bare lignin patches; “Lignin+Fe” denotes lignin patches containing 1 mg Fe g^−1^ lignin in the form of ferrihydrite. **c** The first two principal components of difference spectra marked according to experiment. In experiment 1, liquid media contained 74 µM FeCl_3_•6H_2_O, resulting in mycelia with high overall oxidative decomposition activity, whereas in experiment 2, liquid media did not contain iron, resulting in mycelia with low overall oxidative decomposition activity. **d** The first two principal components of difference spectra marked according to clusters identified using model-based clustering. **e** Average difference spectra for each of the two clusters identified with model-based clustering. Lines above and below each measured wavenumber represent standard deviations (*n* = 92 for cluster 1 and *n* = 76 for cluster 2). Absorbance values equal to 0 indicate no difference in absorbance between hypha spectrum and background spectrum. Positive absorbance values indicate relative increases in absorbance values caused by the presence of hyphae and negative values indicate relative decreases in absorbance values caused by oxidative decomposition of lignin. In the inset, the spectral region from 1480 cm^−1^ to 1520 cm^−1^ is enlarged. Error bars were removed from the inset for clarity. Negative absorbance values in this range are indicative for oxidative lignin decomposition. Absorbance values are expressed in arbitrary units (a.u.). **f** Summary of the number of hyphae with high or low metabolic activity for each patch type and experiment.

Co-varying wavenumbers in difference spectra were converted into PCs to reduce the dimensionality of the data and visualize the variation in difference spectra (Fig. 2a–d). The first two PCs captured a large proportion of the total variation present in difference spectra, indicating that spectral differences at many of the measured wavenumbers were highly correlated. According to these plots, difference spectra marked according to incubation times did not show any clear clustering patterns (Fig. 2a) and difference spectra indeed did not differ significantly between incubation times (*P* = 0.05). Difference spectra tended to separate along the first PC axis according to the presence or absence of iron in lignin patches (Fig. 2b) and the presence or absence of iron in liquid media (Fig. 2c). Difference spectra were significantly different for both factors (*P* < 0.01). Model-based clustering indicated the existence of two distinct clusters of difference spectra (Fig. 2d). One cluster of difference spectra contained spectra showing strong deviations from the original lignin background spectra, whereas the other cluster contained spectra resembling the original lignin spectra more closely (Fig. 2e).

### Chemical modifications of lignin resulted from metabolic activity of *P. involutus* hyphae

The most obvious spectral change induced by hyphae was a strong increase in absorption in the 1000–1200 cm^−1^ range (Fig. 2e). Absorption of IR light in this wavenumber range is typically caused by vibrational modes of bonds found in sugars and sugar-like molecules [37]. The increase in absorbance at these wavenumbers can therefore best be explained by the secretion of sugar-like molecules, such as those commonly found in the extracellular polymeric substances (EPS) secreted by many fungi [38]. Spectral increases in this “sugar region” were sometimes more pronounced at the very tips of hyphae (Supplementary Fig. 2), matching the idea that most active secretion occurs at the apical end of fungal cells [39]. The fungal hyphae themselves could be easily distinguished from the lignin background due to the strong absorption of IR light around 1655 cm^−1^ (Fig. 2e). Absorption of IR light at these wavenumbers results from molecular vibrations of C = O in protein-peptide bonds [40]. The absorption of IR light at 1510 cm^−1^ can be assigned to C = C vibrations of aromatic rings in lignin [41]. Relative decreases in this spectral band in difference spectra are indicative of the breaking of aromatic C = C bonds in lignin and thus of decomposition of lignin by *P. involutus* (Fig. 2e inset). The relative increases in remaining wavenumber regions are more difficult to assign unequivocally and could result from the secretion of secondary metabolites or other unidentified compounds. From these data, we conclude that the two data clusters identified before, using model-based clustering, corresponded to a metabolically highly active cell population (secreting more EPS-like substances and causing more pronounced decomposition of lignin) and a metabolically less active cell population. Interestingly, within a single mycelium, even neighboring hyphae could have contrasting metabolic activity. Outside of hyphae, a decomposition zone was frequently observed. These zones showed spectral changes similar to the changes observed where fungal cells were growing, but spectral deviations from lignin were less pronounced (Supplementary Fig. 3b).

### Relationship between metabolic activity of individual cells and the presence or absence of iron in lignin patches or the overall oxidative decomposition activity of mycelia

Of the metabolically more active cells, most had been growing on lignin patches amended with ferrihydrite, whereas the majority of less active cells had been growing on bare lignin patches without iron (Fig. 2f). Using Fisher’s exact tests, it was concluded that the ratios of hyphae belonging to either the metabolically more or less active cell population were significantly related to the iron content in lignin patches they colonized (*P* < 0.01), irrespective of the overall oxidative decomposition activity of mycelia. Yet, the overall oxidative decomposition activity of mycelia affected the metabolic activity of individual hyphae as well. When iron was present in both the liquid media and lignin patches, the vast majority of hyphae (93%) showed high metabolic activity, whereas when iron was not present in liquid media but present in lignin patches, the ratio of hyphae with high metabolic activity (62%) to less active ones (38%) was more balanced (Fig. 2f). Based on these observations and the overall statistically significant effect presence or absence of iron in liquid media had on difference spectra, we conclude that the effect of the local environment in the lignin patches on single-cell activity was further mediated by the overall oxidative decomposition activity of *P. involutus* colonies.

## Discussion

In the current work, we show that the local environment experienced only by parts of fungal mycelia strongly affects the metabolic activity of individual hyphal tips, determining the outcome of decomposition. This local environmental effect, however, is further mediated by the overall decomposition activity of mycelia. Hence, to better understand the functioning of fungal mycelia in complex environments such as soils, we need to be able to study the chemical interactions between individual fungal cells and their microenvironments.

A number of techniques have become available over the past few decades that allow detailed studies of the chemical interactions at the interface between microbial cells and their environment, including nanoscale secondary ion mass spectrometry, scanning transmission X-ray microscopy, neutron imaging, X-ray fluorescence spectroscopy, X-ray computed tomography, scanning electron microscopy coupled to energy-dispersive X-ray spectroscopy, IR and Raman spectroscopy and fluorescence spectroscopy [16–22]. Among these, IR spectroscopy is currently one of the most suitable tools to investigate the chemical interactions between microbial cells and their environments thanks to the detailed spectral information that can be obtained on organic compounds at a micrometer spatial resolution under ambient conditions [42]. Using modern focal plane-array detectors, entire hyperspectral images can be routinely collected in a matter of minutes. This allows large numbers of hyperspectral images to be collected in a relatively short time, enabling the detailed characterization of the vast diversity and heterogeneity of microbe-environment interactions in a statistical way.

A few technical difficulties, however, need to be taken into consideration when using IR spectroscopy, especially when using this technique to study microbial cells. IR spectroscopy is sensitive to IR absorption due to rotational vibrations of water vapor and CO_2_ gas in the atmosphere [43]. These atmospheric interferences are often counteracted by purging the sample compartment with dry air or N_2_ gas, but spectral artifacts due to IR absorption of water vapor and CO_2_ gas can still be commonly identified in spectra. Another common artifact in spectra collected from microbial cells is caused by Mie scattering [44]. Mie scattering is caused by the wavelength-dependent diffraction of IR light by objects with different optical densities in samples [45]. These spectral artifacts need to be reduced as much as possible to obtain pure absorption spectra of the sample under investigation, otherwise spectra may be misinterpreted. In the current study, both artifacts due to atmospheric gasses and Mie scattering were minimized using in-house built software.

To make comparisons across many hyperspectral images, the substrate thickness and uniformity must be tightly controlled as well. Spin-coating of organic polymers such as lignin, hemicellulose, proteins, etc. was found to be the most suitable method of all techniques explored, producing uniform organic patches with only nanometer thickness variations. The production of suitable cellulose patches still needs further development as there are currently no volatile, nonderivatizing solvents known for cellulose. To investigate the chemical interactions between microbes and soil samples, a method to produce thin sections of soils needs to be developed. Routine preparation of thin soil sections requires embedding soil into a resin, which introduces chemical changes in the soil sample; furthermore, the mixture of harder and softer materials in soils prevents thin sections being of uniform thickness [46].

In the current study, hyphae from the same colony exposed to the same environment were shown to still be heterogeneous in their metabolic activity, even for neighboring hyphae. Hyphal phenotypical heterogeneity within colonies has been documented in the past for the ascomycetes *Aspergillus oryzae* [47] and *A. niger* [14, 48–50] and for the Basidiomycete *Schizophyllum commune* [51]. In case of *A. niger*, 27% of hyphae in a colony showed low expression profiles of a glucoamylase gene (glaA), whereas the remainder of the hyphae showed higher expression profiles [14]. A similar, bimodal, distribution of actively decomposing versus less metabolically active hyphae was observed in the current study.

We also show that increased decomposition activity in fungi may be the result of a larger number of hyphae in a mycelium participating in decomposition, rather than an overall increase in the decomposition activity of all hyphae in a mycelium. This observed hyphal heterogeneity may have evolved as an adaptation in fungi for foraging in heterogeneous and constantly changing environments such as soils. In *Saccharomyces cerevisiae*, phenotypic switching rates between individual cells have indeed been linked to the rate at which the environment changes [52], but a link between hyphal phenotypic heterogeneity and the heterogeneous nature of soils has yet to be established. It is currently unclear how fungal mycelia integrate information coming from hyphae inside and outside of nutrient patches. Nutrient concentrations inside mycelia may be one way by which signals are transduced throughout mycelia as it is clear that nutrients can be transported towards or away from the growing hyphal front, depending on the nutritional needs of different parts of a mycelium [12, 53, 54].

A number of mechanisms may underlie hyphal metabolic heterogeneity in filamentous fungi. A first mechanism involves the dynamic closure of septa (through Woronin bodies in Ascomycetes or parenthesomes in Basidiomycetes). Woronin bodies originate from peroxisomes through budding and serve to plug damaged hyphae or stop cytoplasmic streaming through septa [5]. The closure of septa through Woronin bodies has been shown to maintain different levels of gene expression in *A. oryzae* hyphae [47–50]. Moreover, dynamic closure and opening of septa was recently found to induce directional flow of cytoplasm in individual hyphae of the Basidiomycete *Coprinopsis cinerea* [53]. In that study, the net bulk movement of cytoplasm was shown to alternate between basipetal and acropetal directions every 2–3 h, where different cells of the same colony can exhibit different flow directions simultaneously. Another factor that may induce temporal variation in metabolic activity of different cells in a mycelium is the different stages of the cell cycle that hyphae may be in at any given time [55]. It is clear that hyphal tip growth and exocytosis of new cellular building blocks occur in bursts rather than in a continuous fashion in filamentous fungi [56]. Hence, the secretion of EPS, enzymes and secondary metabolites could follow similar discrete activity patterns. Finally, there are a number of molecular mechanisms that may generate and maintain phenotypic heterogeneity in isogenic cells. These molecular mechanisms include stochastic gene expression, stochastic partitioning of molecules at cell division and epigenetic modifications of chromatin through DNA methylation and/or histone composition [57].

The presence of a strong absorption signal for EPS-like compounds in the hyphae that also showed the most pronounced lignin decomposition (Fig. 2e) suggests the involvement of the EPS in the oxidative decomposition of lignin. Interestingly, the involvement of EPS in wood decomposition by the brown-rot fungus *Serpula lacrymans* [58] and the white-rot fungi *Phanerochaete chrysosporium* [59] and a number of *Pleurotus* species [60] has been suggested in the past. These studies investigated and discussed a number of possible roles for EPS during wood decomposition: (i) EPS may create a suitable environment for decomposition by facilitating diffusion and concentrating various biodegrading enzymes. (ii) Fungi may also use the EPS layer to generate gradients of pH and ionic strength to promote the production of highly oxidizing ^•^OH away from the fungus, while protecting their own cell walls. (iii) The presence of polysaccharides in the EPS can further protect fungal cells from oxidative damage by acting as radical scavengers.

## Conclusions

We developed a procedure based on IR spectroscopy that allows the in-situ, nondestructive hyperspectral imaging of chemical interactions between individual fungal hyphal tips and their immediate environments. We show that the local environment experienced only by parts of mycelia in discrete patches plays an important role in driving fungal decomposition activity, but that this effect is further mediated by the overall decomposition activity of mycelia. Increased fungal decomposition may be the result of a larger proportion of hyphae in a mycelium that participate in decomposition, rather than an overall increase in decomposition activity of all hyphae in a mycelium. Even at the single-cell level, decomposition was shown to be heterogeneous, with decomposition zones extending beyond hyphae and with hyphal tips showing increased decomposition activity. Spectra also suggested a role for the EPS produced by *P. involutus* in decomposition. The observation that the decomposition activity is largely regulated by the conditions in the local environment may explain why physical and chemical soil parameters measured in bulk samples do not correlate well with microbial decomposition activity [15].

## Supplementary information

Supplementary Figure 1

Supplementary Figure 2

Supplementary Figure 3
