# Supplementary material for: Regulation of fungal decomposition at single-cell level
Source: ISME J. 2020 Jan 2;14(4):896–905. doi: 10.1038/s41396-019-0583-9 (PMC7082364; doi:10.1038/s41396-019-0583-9)
Supplement: Supplementary file 1 — Supplementary Figure 1 [file 41396_2019_583_MOESM1_ESM.pdf]

Supplementary Figure 1

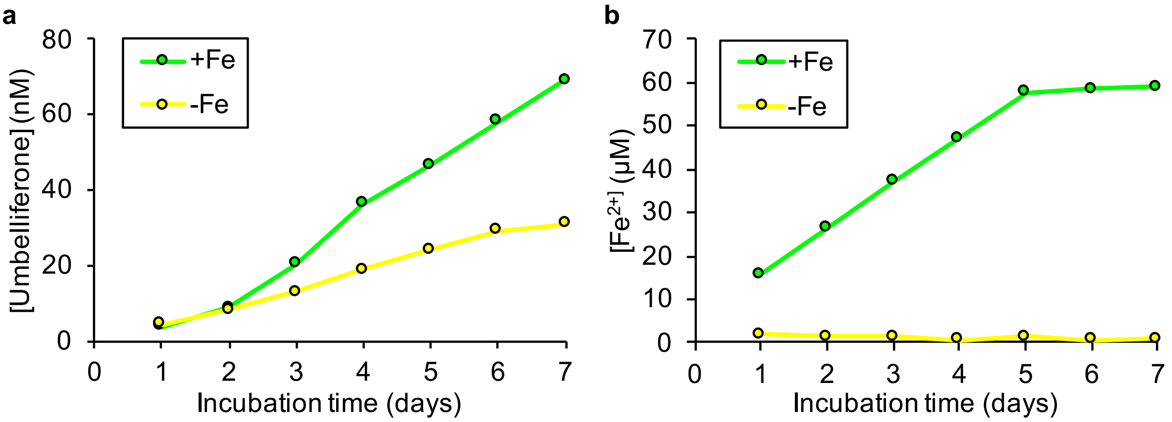

Supplementary Fig. 1. Overall oxidative decomposition activity of *Paxillus involutus* colonies growing in the presence or absence of iron in liquid growth media. **a** Production of hydroxyl radicals by *P. involutus* in the presence of iron in the liquid growth medium results in the oxidation of non-fluorescent coumarin to fluorescent umbelliferone which is subsequently measured with a fluorescence spectrometer. See [1] for more details on the method used to detect the production of hydroxyl radicals. In the absence of iron in the growth medium, the production of hydroxyl radicals is much reduced, resulting in mycelia with low overall oxidative decomposition activity. In the presence of iron in liquid media, much higher concentrations of hydroxyl radicals are detected, indicating that mycelia have a much more pronounced oxidative decomposition activity under these conditions. **b** Most of the iron present in liquid growth media is reduced by *P. involutus*. No reduced iron could be detected when no iron was supplied in the liquid growth media. See [1] for the method used to measure reduced iron concentrations.

References

1. Op De Beeck M, Troein C, Peterson C, Persson P, Tunlid A. Fenton reaction facilitates organic nitrogen acquisition by an ectomycorrhizal fungus. New Phytol. 2018; 218: 335-343.
