# Supplementary material for: Regulation of fungal decomposition at single-cell level
Source: ISME J. 2020 Jan 2;14(4):896–905. doi: 10.1038/s41396-019-0583-9 (PMC7082364; doi:10.1038/s41396-019-0583-9)
Supplement: Supplementary file 2 — Supplementary Figure 2 [file 41396_2019_583_MOESM2_ESM.pdf]

Supplementary Figure 2

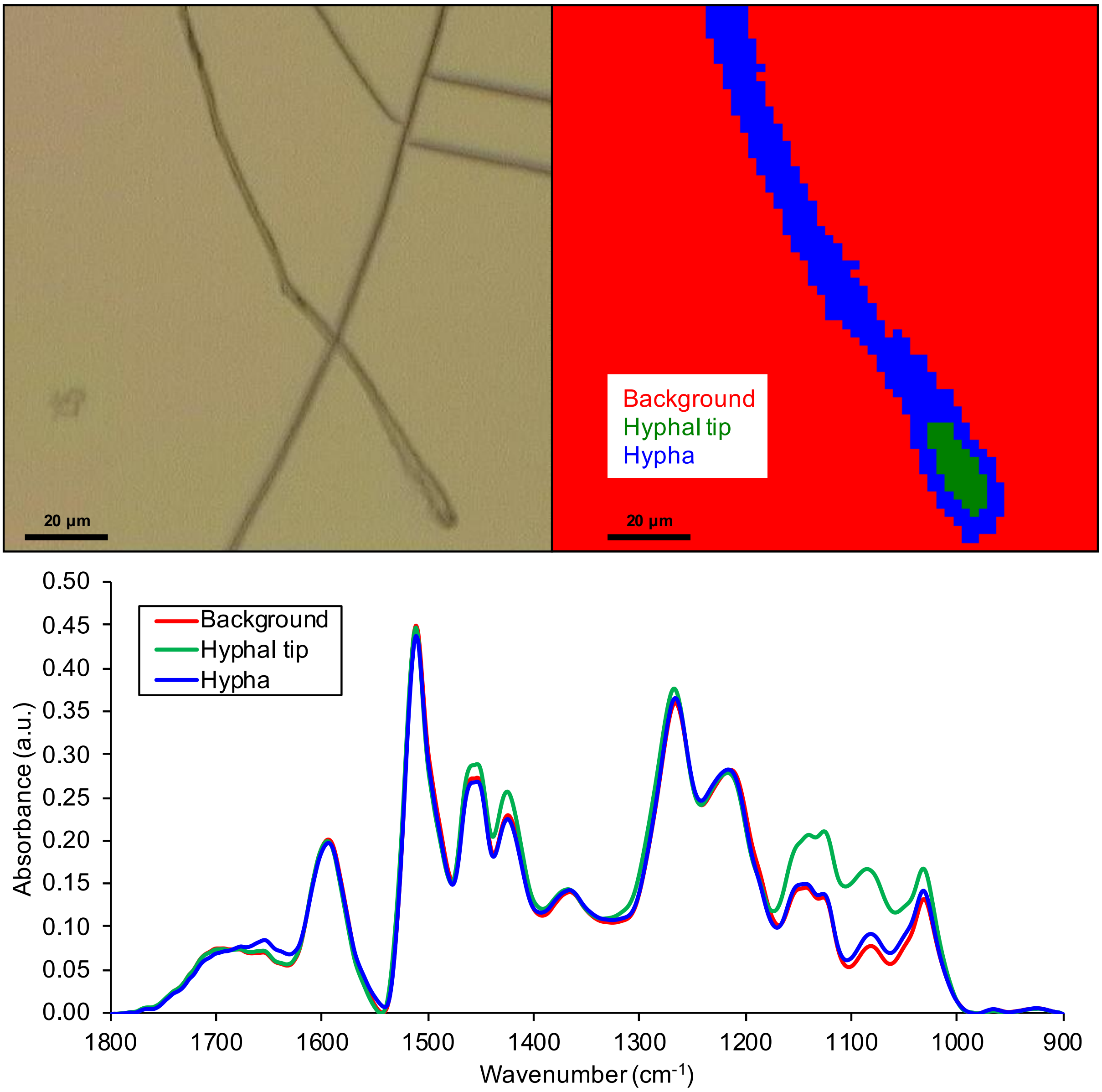

Supplementary Fig. 2. Hyperspectral image of a *Paxillus involutus* hypha colonizing a lignin patch, showing increased oxidative decomposition activity at the hyphal tip. A discrete cluster, as detected by the chemical imaging procedure, can be recognized (green cluster) at the hyphal tip where spectral changes in the lignin substrate are more pronounced (green spectrum) compared to the spectral changes induced by the remainder of the hypha (blue spectra). The top left image is the white light image of the selected hypha. The top right image is the result from cluster analysis in the chemical imaging procedure for the same hypha. The bottom image displays the average infrared absorbance spectra for each cluster.
