# Supplementary material for: Regulation of fungal decomposition at single-cell level
Source: ISME J. 2020 Jan 2;14(4):896–905. doi: 10.1038/s41396-019-0583-9 (PMC7082364; doi:10.1038/s41396-019-0583-9)
Supplement: Supplementary file 3 — Supplementary Figure 3 [file 41396_2019_583_MOESM3_ESM.pdf]

Supplementary Figure 3

a

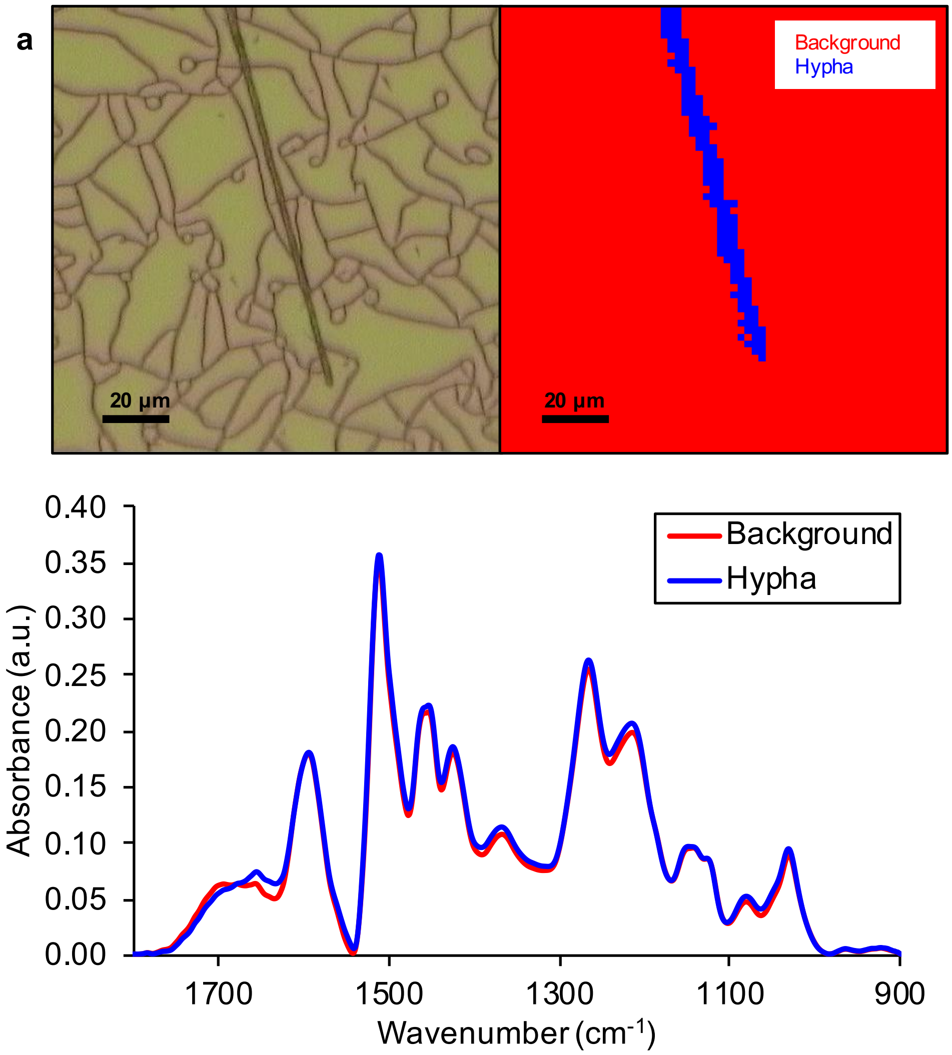

b

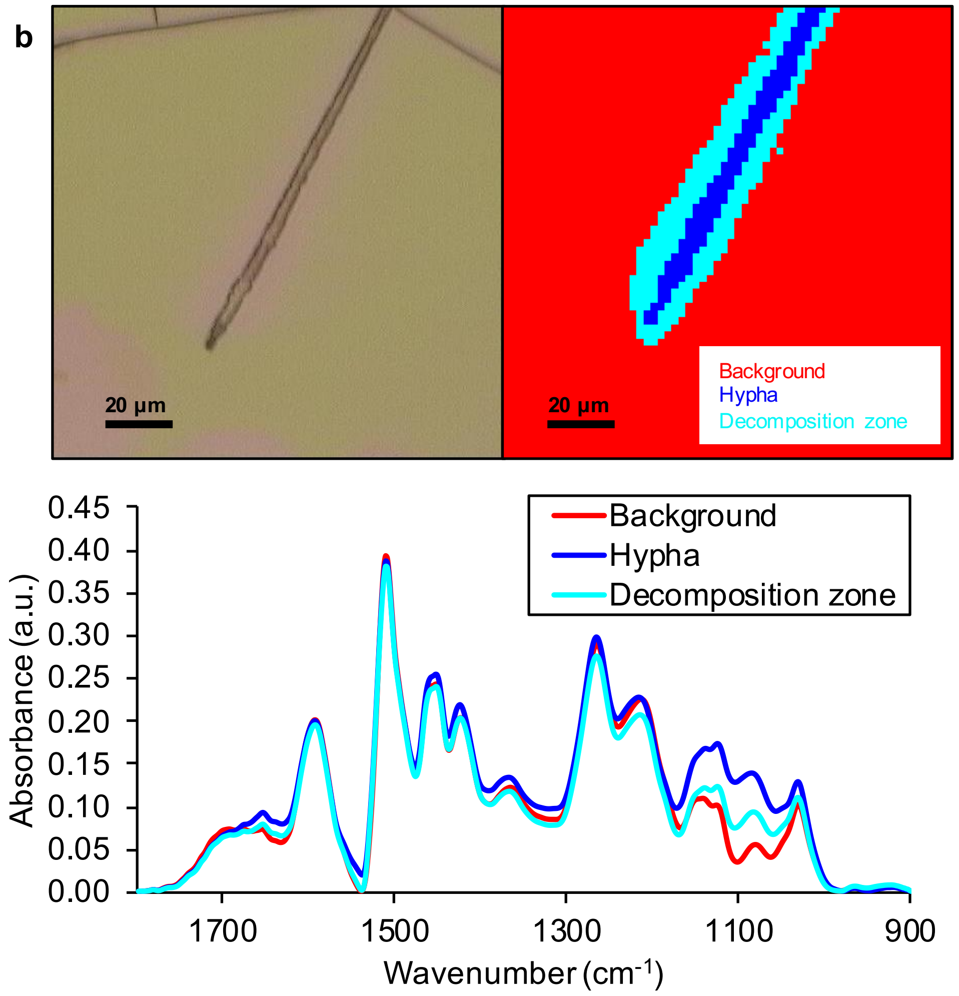

Supplementary Fig. 3. Hyperspectral images of *Paxillus involutus* hyphae colonizing lignin patches, showing an inactive hypha and a hypha actively decomposing the lignin substrate. **a** Example of a *P. involutus* hypha colonizing a lignin patch without actively decomposing the lignin. The relative increase in the peak at 1655 cm^{-1} is due to the presence of proteins in the hypha. **b** Example of a *P. involutus* hypha colonizing a lignin patch and causing substantial chemical changes in the substrate. A distinct decomposition zone around the hypha can be identified as well. The decomposition zone in this image measures 2 µm on its most narrow side and 8 µm on its widest side. Top left images in panels are white light images of respective hyphae. Top right images in panels are results from cluster analyses from the chemical imaging procedure for corresponding hyphae. Bottom images of panels display average infrared absorbance spectra for each cluster.
